# Biologically relevant small variations of intra-cellular pH can have significant effect on stability of protein–DNA complexes, including the nucleosome

**DOI:** 10.3389/fmolb.2023.1067787

**Published:** 2023-04-18

**Authors:** Alexey V. Onufriev

**Affiliations:** ^1^ Department of Physics, Virginia Tech, Blacksburg, Blacksburg, VA, United States; ^2^ Department of Computer Science, Virginia Tech, Blacksburg, Blacksburg, VA, United States; ^3^ Center from Soft Matter and Biological Physics, Virginia Tech, Blacksburg, VA, United States

**Keywords:** protein DNA complex, stability, binding affinity, pH, nucleosome, DNA accessibility, transcription

## Abstract

Stability of a protein-ligand complex may be sensitive to pH of its environment. Here we explore, computationally, stability of a set of protein-nucleic acid complexes using fundamental thermodynamic linkage relationship. The nucleosome, as well as an essentially random selection of 20 protein complexes with DNA or RNA, are included in the analysis. An increase in intra-cellular/intra-nuclear pH destabilizes most complexes, including the nucleosome. We propose to quantify the effect by ΔΔG^0.3^—the change in the binding free energy due to pH increase of 0.3 units, corresponding to doubling of the *H* ^+^ activity; variations of pH of this amplitude can occur in living cells, including in the course of the cell cycle, and in cancer cells relative to normal ones. We suggest, based on relevant experimental findings, a threshold of biological significance of $\frac{1}{2}k_{B}T$ (∼0.3kcal/mol) for changes of stability of chromatin-related protein-DNA complexes: a change in the binding affinity above the threshold may have biological consequences. We find that for 70% of the examined complexes, ${\Delta \Delta G}^{0.3}\;>\frac{1}{2}k_{B}T$ (for 10%, ΔΔG^0.3^ is between 3 and 4 *k*_*B*_ *T*). Thus, small but relevant variations of intra-nuclear pH of 0.3 may have biological consequences for many protein-nucleic acid complexes. The binding affinity between the histone octamer and its DNA, which directly affects the DNA accessibility in the nucleosome, is predicted to be highly sensitive to intra-nuclear pH. A variation of 0.3 units results in ΔΔG^0.3^ ∼ 10*k*_*B*_ *T* (∼6kcal/mol) ; for spontaneous unwrapping of 20 bp long entry/exit fragments of the nucleosomal DNA, ΔΔG^0.3^ = 2.2*k*_*B*_ *T*; partial disassembly of the nucleosome into the tetrasome is characterized by ΔΔG^0.3^ = 5.2*k*_*B*_ *T*. The predicted pH -induced modulations of the nucleosome stability are significant enough to suggest that they may have consequences relevant to the biological function of the nucleosome. Accessibility of the nucleosomal DNA is predicted to positively correlate with pH variations during the cell cycle; an increase in intra-cellular pH seen in cancer cells is predicted to lead to a more accessible nucleosomal DNA; a drop in pH associated with apoptosis is predicted to make nucleosomal DNA less accessible. We speculate that processes that depend on accessibility to the DNA in the nucleosomes, such as transcription or DNA replication, might become upregulated due to relatively small, but nevertheless realistic increases of intra-nuclear pH.

## 1 Introduction

The nucleus of a eukaryotic cell contains its DNA bound to various proteins, surrounded by an atmosphere of diverse counter-ions (Korolev et al., 2007). This giant protein-DNA complex, referred to as “chromatin (Misteli, 2007),” is central to many of the most important cellular processes such as cell differentiation, DNA replication, repair, transcription, and epigenetic inheritance, *i.e.,* inheritance that is not coded by the DNA sequence (Henikoff, 2008). The “hydrogen atom” of chromatin is the nucleosome (Woodcock, 1973; Kornberg, 1974; Olins and Olins, 1974)—the primary, fundamental level of the DNA compaction (Onufriev and Schiessel, 2019). The nucleosome core particle (Luger et al., 1997), (H2A⋅H2B)_2_⋅ (H3⋅H4)_2_ ⋅ DNA, which we refer to as the nucleosome for simplicity, consists of 147 base pairs of DNA tightly wrapped around a protein core made of two copies of each of the four histone proteins H2A, H2B, H3, H4. Chromatin compaction at the nucleosome level is believed to be the most relevant to gene access, recognition (Misteli, 2007) and regulation (Kornberg and Lorch, 2020). The strength of the protein-DNA association in the nucleosome affects accessibility (Onufriev and Schiessel, 2019) of the nucleosomal DNA to cellular machines that need access to its information content; changes in the DNA accessibility can have direct biological consequences. For example, small increases in nucleosomal DNA accessibility (Onufriev and Schiessel, 2019) can lead to major increases in steady-state transcript levels (Zhu and Thiele, 1996) and promoter activity (Raveh-Sadka et al., 2012). The state of the nucleosome and chromatin, and thus access to its DNA, can be modulated in various ways. One example is reversible structural modifications to the histone proteins (Tessarz and Kouzarides, 2014) such as acetylation, in which normally positively charged Lysine groups become neutralized, generally leading to decreased histone-DNA binding affinity and the more accessible DNA (Fenley et al., 2010; Fenley et al., 2018). Another example is spontaneous and transient unwrapping of the nucleosomal DNA fragments at each end (Gansen et al., 2009; Koopmans et al., 2009; Tims et al., 2011), the corresponding life-times of these partially unwrapped states may be just long enough for functionally relevant access to DNA target sites located there (Tims et al., 2011). Another mechanism that can enhance access to the nucleosomal DNA is progressive disassembly of the histone octamer itself (Zlatanova et al., 2009; Andrews and Luger, 2011; Luger et al., 2012; Kato et al., 2017), which leads to the formation of partially assembled nucleosome structures (PANS), each lacking several histone proteins (Rychkov et al., 2017). For example, in the tetrasome, (H3⋅H4)_2_ ⋅ DNA, about 78 bp of the DNA is accessible (Rychkov et al., 2017). The tetrasome is a key intermediate on the nucleosome assembly/disassembly pathway (Andrews and Luger, 2011), including during DNA replication (Zhang et al., 2020). One of the central open questions is exactly how DNA accessibility is controlled, at the level of the nucleosome? Many such mechanisms have been determined, while probably just as many remain to be uncovered. Could variations in pH be one of them?

A number of other protein-DNA complexes are just as vital to chromatin function, including transcription factors and chromatin remodeling complexes. Their specific, high enough, binding affinity for the DNA is critical for the function of chromatin. In general, modulation of the strength (free energy) of protein-DNA association may be expected to have biological consequences if these free energy changes are comparable to other relevant free energy scales in the system. The main question we address in this work is whether biologically relevant variations in ambient pH can bring about large enough changes in the protein-DNA binding free energy to be of potential relevance to chromatin function.

Many key processes in living cells are sensitive, *via* a diverse set of mechanisms, to the pH of the medium where the process takes place (Parker and Boron, 2013). In particular, macromolecular complexes are key players in a myriad of cellular functions: if properties of such a complex depend on pH, one can reasonably expect its function to be also affected by pH. Within a given cell, depending on the specific organelle, macromolecular complexes can be exposed (Chan et al., 2006; Garcia-Moreno, 2009; Kulichikhin et al., 2009) to a wide range of pH from about 5 to 8.

Multiple experimental and computational studies have demonstrated definitively that stability of protein-protein complexes tend to depend on pH, often over a wide range; the many associated nuances and biological consequences have been investigated and discussed in detail, see, e.g., Ref (Kundrotas and Alexov, 2006) and multiple references therein. In contrast, relatively less is firmly established with respect to pH dependence of protein-nucleic acid complexes, especially for those relevant to chromatin function. For the nucleosome, experiment demonstrated (Libertini and Small, 1984) that the structure becomes looser upon pH increase from about 7 to 8, consistent with the notion that the histone-DNA binding depends on pH. Unfortunately, we aren’t aware of relevant measurements or predictions directly reporting *quantitative* data on pH dependence for the nucleosome; without these, it is hard to conclude whether the corresponding pH effects are biologically significant over the small pH variations expected within the nucleus, where the complex is found. For protein - nucleic acid complexes in general, an earlier theoretical work (Record et al., 1978) concluded that free energy of protein-DNA interactions should decrease with pH. We are aware of only a few relevant experimental data points (Senear and Ackers, 1990; Wessel et al., 1992; Peng and Acheson, 1998; Torigoe et al., 2003; Hamimes et al., 2006; Deegan et al., 2010; Moreau et al., 2012)—these show consistently that complex stability decreases with pH, however, most of the conclusions are qualitative, the strength of the reported effect varies substantially from study to study. Reported computational predictions also vary with respect to the significance of pH dependence or, equivalently, net proton uptake, in protein - nucleic acid binding. A fairly strong complex destabilization with increasing pH, consistent with experiment, was predicted for the *λ*-repressor (Misra et al., 1998). On the other hand, a recent large-scale computational study (Peng and Alexov, 2017) concluded that very little net proton uptake/release occurred upon binding between proteins and nucleic acids between pH = 5 to 8. We are unaware of similar studies focusing specifically on chromatin-related protein-DNA complexes, or on the nucleosome and its partially assembled and partially unwrapped states. This work is intended to fill the gap, focusing on quantitative predictions suggestive of possible biological relevance.

Note that while intra-cellular and intra-nuclear pH is tightly controlled by the cell (Garcia-Moreno, 2009; Parker and Boron, 2013), variations do occur as part of the normal cell function, as well as in various pathologies such as cancer (Parker and Boron, 2013). Specifically, intra-cellular pH varies along the cell cycle (White et al., 2017), with a transient increase from 7.2 to 7.6. In mutant cells in which this increase in pH is attenuated, S phase is delayed, and G2/M transition is impaired (Putney and Barber, 2003), suggesting that pH variation may play a role in the cell cycle progression. Intra-cellular acidification by as much as 1 pH unit was observed to precede apoptosis, and was proposed as a possible effector of the apoptosis program (Gottlieb et al., 1995). In cancer cells, intra-cellular pH is increased by about 0.4 units compared to normal cells (while extra-cellular pH is decreased) (White et al., 2017). Some of these small changes in pH have been shown to correlate with vital cellular processes, likely involving chromatin components. For example, in higher eukaryotes, increases in the intra-cellular pH have been shown to correlate with cell proliferation and differentiation (Orij et al., 2011). In breast cancel cells, cell cycle progression is proposed to involve cell cycle phase-specific pH regulation (Flinck et al., 2018).

While a computational work such as this one can not firmly establish a causality link from a relevant pH variation to biological function, it can attempt to predict whether or not the associated pH induced changes in the binding affinity of at least some of chromatin-related protein-nucleic acid complexes are large enough to be potentially consequential, or whether these changes are non-existent or too small to consider seriously. If these changes are large enough, by a certain set of criteria that we set out to formulate, then predictions can be put forward, follow-up experiments can be suggested, and intriguing speculations can be made regarding possible effect of pH variations on biological function. This study aims to carry out the program just outlined.

We conclude this section by introducing some of the relevant terminology and concepts. Investigation of pH dependence of complex formation, either experimental or computational, is aided by the so-called linkage relationship: it follows from basic thermodynamics (Tanford, 1970) that whenever a net proton uptake/release occurs upon complex formation between the ligand and the receptor, the corresponding binding free energy, that is complex stability, depends on pH of the environment. The proton uptake/release is directly related to binding-induced changes (shifts) in p*K* ^1^ values of titratable (ionizable) groups in proteins and their ligands. Since micro-environment of many titratable groups changes upon complex formation, their p*K* values can be expected to change in response; the changes can be predicted by a variety of well-established computational methods (Bashford and Karplus, 1990; Yang et al., 1993; Antosiewicz et al., 1994; DelBuono et al., 1994; Demchuk and Wade, 1996; Sham et al., 1997; Ullmann and Knapp, 1999; Nielson and Vriend, 2001; Georgescu et al., 2002; Mongan et al., 2004; Khandogin and Brooks, 2006; Bas et al., 2008; Kieseritzky and Knapp, 2008; Spassov and Yan, 2008; Song et al., 2009; Huang et al., 2018; Pahari et al., 2018). Indeed, p*K* shifts in protein-ligand binding, sometimes significant (Aguilar et al., 2010) (|ΔpK | > 1), are well-documented, both experimentally and computationally, see, e.g*.*, these reviews (Onufriev and Alexov, 2013; Petukh et al., 2013); several distinct physical mechanisms are behind these shifts, including electrostatic (Zhang et al., 2011) and “allosteric” (Aguilar et al., 2010). Importantly, p*K* shifts induced by ligand binding are only necessary, but not sufficient (Onufriev and Alexov, 2013) for net proton release/uptake required for pH dependence of the binding affinity, which makes the question of whether or not a noticeable pH dependence exists a more subtle one than simply determining if meaningful p*K* shifts occur upon binding.

## 2 Material and methods

We begin, Section. 2.1, with a description of the over-all strategy for selecting the atomistic structures used in this work, and their initial preparation. In the following subsections, 2.1.1, 2.1.2, 2.1.3, we present detailed descriptions of each set of structures, starting from smaller protein - nucleic acid complexes, followed by the nucleosome and its partially unwrapped and partially assembled states. Next, in Section. 2.2, we describe the computational protocol employed to estimate p*K* s (titration curves) of the relevant titratable groups, and the pH dependence of the net charge of each complex. The results of these calculations are used to estimate pH dependence of the complex stability, as described in the final Section. 2.3.

### 2.1 Structure selection and initial preparation

When available, separate, independent experimental structures for the complex and the unligated (apo) protein were used. This protocol, which explicitly accounts for binding-induced structural re-arrangements in the protein, was applied to the 20 protein-nucleic acid complexes described in Section. 2.1.1. When the corresponding unligated structure of the protein was unavailable, an alternative protocol was used: the unligated protein was constructed from the structure of the complex, by manually separating the nucleic acid from the protein in the PDB file. This protocol was applied to all other complexes described in this work. Limitations of the latter approximation, which does not account for binding-induced structural re-arrangements, are well recognized (Alexov et al., 2011); however, alternatives such as constant pH MD (Wallace and Shen, 2009; Swails et al., 2014), which could mitigate the issue, are still too expensive for large complexes such as the nucleosome. Unless otherwise stated, the structures used as input for p*K* calculations, Section 2.2 below, were not manipulated beyond the steps described above.

#### 2.1.1 20 protein-nucleic acid complexes

We have employed the same set of 20 protein-nucleic acid complexes that were previously used by us for an analysis of p*K* shifts in protein-ligand binding in Ref. (Aguilar et al., 2010); the detailed selection criteria, structure preparation procedures can be found *ibid*, a brief description is below. The PDB IDs are given in Supplementary Table S1 of the Supplementary Information.

The structures were selected out of 1932 entries available at the time (Aguilar et al., 2010) in NPIDB database (Spirin et al., 2007) of protein-nucleic acid complexes. Since accuracy of p*K* calculation and protonation state assignment depend critically on the quality of the input structure, the 20 highest quality structures were chosen that satisfy the following three criteria: 1) no missing residues; 2) 2.5 Å or better resolution; 3) availability of high quality structures for *both* the complex and the unligated protein, as separate PDB entries. These strict selection criteria explain the relatively small size of the set. It also makes the selection essentially random (20 out of near 2000).

The selected complexes represent at least 14 different functional classes; the ligand is DNA in 10 cases, RNA in 9, and in one case the ligand is an RNA/DNA hybrid, see Supplementary Table S1 of the Supplementary Information. The proteins in the complexes vary widely with respect to the number of titratable groups: from 18 to 264.

#### 2.1.2 The lambda repressor complex

The 1.8 Å resolution structure of the *λ* repressor complex was used, PDB ID: 1LMB. To the best of our knowledge, no high resolution atomistic structure of the corresponding unligated protein is available for this complex, therefore the unligated structure was prepared manually, as described in Section 2.1. The relevant pH -related experimental data points are from Table 3 of Ref. (Senear and Ackers, 1990), corresponding to *O*_*R*_1; the specific choice is based on the argument presented in Ref. (Misra et al., 1998). The pH dependence of the *λ* repressor stability is particularly well characterized experimentally, making it a unique reference for a computational study such as this one.

#### 2.1.3 The nucleosome and its partially unwrapped/assembled states

##### 2.1.3.1 The canonical nucleosome

The following PDB structures were used: 5B0Z 1.99 Å (*homo sapiens*), 1AOI, 2.8 Å (*xenopus laevis*), 1KX5 1.9 Å (chimeric). To the best of our knowledge, no atomistic structures of the “bare” histone core is available, therefore the unligated structures of the nucleosome, as well as of its derivatives described below, were prepared manually, by removing the appropriate DNA fragments from the complex, Section 2.1.

##### 2.1.3.2 Partially unwrapped state of the nucleosome

**FIGURE 1:**
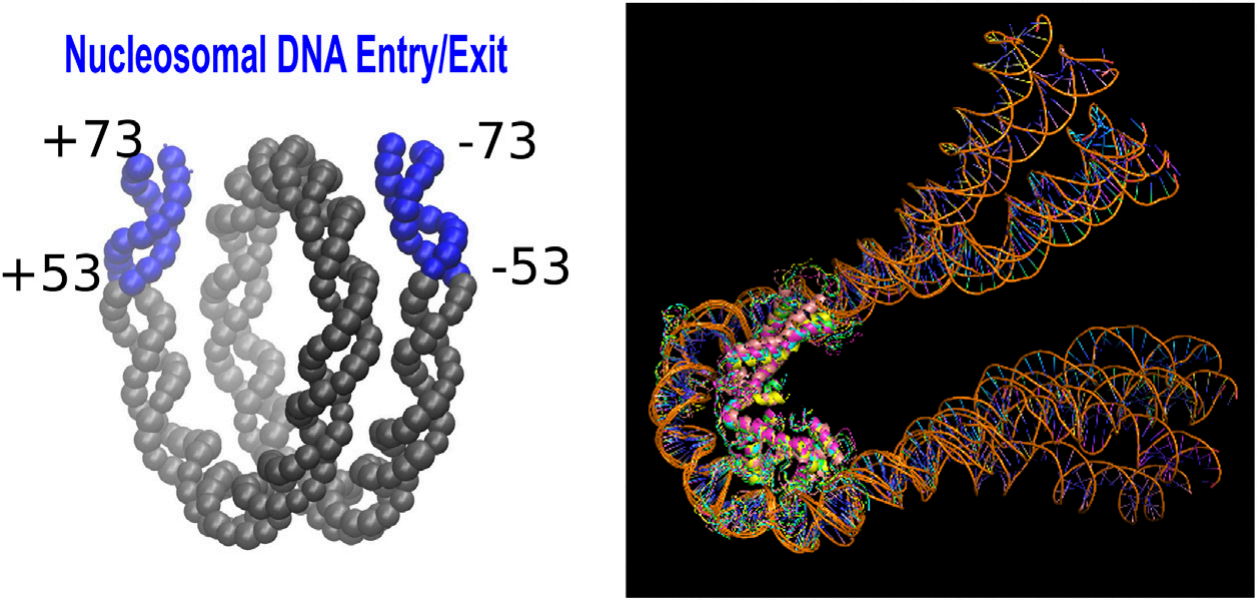
Left: A schematic showing the Entry/Exit segments (20 base pairs at each end) of the nucleosomal DNA (Fenley et al., 2018). This region becomes exposed in the partially unwrapped state of the nucleosome. Right: The structural ensemble representing (Rychkov et al., 2017) the tetrasome.

The partially unwrapped state of the nucleosome, PW(20.20), in which 20 bp on each end “peel off” the histone core was approximated as follows. We removed the corresponding DNA fragments, blue regions in Figure 1 (left), from 1KX5 structure, keeping the DNA in positions from −52 to +52. The computed ΔΔG^0.3^ characterizes the transition *PW*(20.20) + 40*bp* → *nucleosome*.

##### 2.1.3.3 The tetrasome

No experimental structure of the tetrasome, (H3⋅H4)_2_ ⋅ DNA, is available, so we used the one obtained previously by atomistic modeling guided by AFM experiment (Rychkov et al., 2017). To account for non-negligible conformational variability of the tetrasome, we employed models 1,2,3,9, and 10 from Ref (Rychkov et al., 2017).—snapshots from an MD simulation—to represent the tetrasome, Figure 1 (right). These models utilized 1KX5 nucleosome structure (Rychkov et al., 2017) as the basis.

The total charge of the tetrasome complex is the average of the charges obtained for each of the models. The (H2A⋅H2B)_2_ dimer structure was taken from 1KX5 nucleosome structure. The transition considered here is (H3⋅H4)_2_ ⋅ DNA + (H2A⋅H2B)_2_ → *nucleosome*.

### 2.2 Calculation of p*K* values and pH -dependence of net change of the structure

We used H++ server (Gordon et al., 2005; Anandakrishnan et al., 2012), http://biophysics.cs.vt.edu/H++, to prepare the input structures, including assignment of protons. Minimal pre-processing, detailed in Refs. (Gordon et al., 2005; Anandakrishnan et al., 2012), is performed on the input structures; this step includes assignment of missing heavy atoms within existing residues, and initial assignment of missing protons. No missing residues are added: structures with missing residues in the middle of the chain were not considered. The same H++ server was used to compute the p*K* (p*K*_1/2_) values and titration curves of all ionizable residues in the proteins. Version 4.0 was used for individual calculations, except for the 20 protein-nucleic acid complexes for which an in-house (batch) script based on version 3.0 was employed. The calculations were based on the standard continuum electrostatics methodology (Bashford and Karplus, 1990) as implemented in H++ server, proper Boltzmann averages over all protonation states were computed (Beroza et al., 1991; Myers et al., 2006). As is common (Senear and Ackers, 1990; Misra et al., 1998), nucleic acid groups were assumed to have relatively low p*K* values, and therefore treated as non-titratable at neutral pH of interest; a recent study (Peng and Alexov, 2017) showed that their computed p*K* values are shifted down upon protein binding, providing further justification for excluding these groups from our calculations that focus on neutral pH. Unless otherwise stated, the protein and its ligand were treated as a low dielectric medium with the default value for the internal dielectric constant *ϵ*_*in*_ = 10, while the surrounding solvent was assigned a high dielectric constant *ϵ*_*out*_ = 80. The electrostatic screening effects of (monovalent) salt enter *via* the Debye-Huckel screening parameter *κ* = 0.128Å^−1^ which roughly corresponds to a physiological concentration of [NaCl] = 0.15M. Among the input parameters of these calculations, arguably the largest uncertainty is in the choice of *ϵ*_*in*_, its effect is discussed in Section 3.2. The dependence of p*K* values in proteins on the solvent salt concentration in the physiological range is known to be relatively weak (Kao et al., 2000), unlikely to bring about changes in the net proton uptake comparable to those due to changes in pH considered here. In addition to the specific protein- (nucleic acid) experimental data points discussed in detail in “Results,” the methodology used in this work was tested on a larger set of experimental p*K* shifts in protein-protein and protein-small molecule complexes (Aguilar et al., 2010).

### 2.3 Calculation of the pH dependence of the complex stability, Δ*G*(pH)

Once the titration curve of the protein was obtained, and the charge of the structure computed (charge.dat in H++), a general thermodynamic linkage relationship (Tanford, 1970) was be used to estimate the pH -dependent correction to the binding energy:

$$\Delta G\left(pH\;\right)=\Delta G\left({pH\;}_{\mathrm{ref}}\right)+2.3k_{B}T\int_{{pH\;}_{\mathrm{ref}}}^{pH\;}\Delta Q\left(pH\;\right)dpH\;$$ (1)

Where Δ*Q*(pH) is the difference between the net charge of the complex and the corresponding free protein and the ligand at the given pH, Δ*Q*(pH) = *Q*_*complex*_(pH) − *Q*_*unbound*_(pH) = *Q*_*complex*_(pH) − *Q*_*protein*_(pH) − *Q*_*ligand*_(pH). The reference pH_*ref*_ can be chosen arbitrarily, and is specified in the text if relevant. We use *k*_*B*_ *T* = 0.59 *kcal*/*mol* where appropriate.

Another convenient form of the linkage relationship in Eq. 1 is

$$\frac{\partial \Delta G\;}{\partial pH\;}=2.3k_{B}T\Delta Q\left(pH\;\right),$$ (2)

$\frac{\partial \Delta G\;}{\partial pH\;}>0$ means that the complex is destabilized with increasing pH, which obviously requires that the corresponding Δ*Q* > 0.

## 3 Results and discussion

The main objective of this work is to determine whether biologically relevant variations of pH of the environment may cause potentially biologically relevant changes in thermodynamic stability of protein - (nucleic acid) complexes relevant to chromatin function. Our focus is only on ΔG(pH), and its possible biological significance, for chromatin -specific complexes, many other aspects of protein-nucleic acid binding (Luscombe et al., 2001; Lejeune et al., 2005; Rohs et al., 2009; Peng and Alexov, 2017) aren’t explored here. Due to the relative paucity of chromatin specific complexes such as chromatin remodeling and transcription factors among protein - nucleic complex that pass our selection criteria for suitability for p*K* calculations, see Methods, our strategy is as follows. To be able to make statistically significant statements we broaden the pool of complexes to include not only chromatin specific complexes, but several other classes as well. Our justification is that the underlying physics of pH sensitivity of protein-ligand complexes stability is universal (Kundrotas and Alexov, 2006; Peng and Alexov, 2017), and thus our general conclusions made for a diverse set of complexes can serve as a reasonable approximation to the specific class of interest. We recognize that the nucleosome is a special case due to its unique and unusual structure, this complex is investigated separately.

For small variations ΔpH, that is to the first order, it follows from Eq. 2 that the corresponding change of the complex stability:

$$\Delta \Delta G\;\approx 2.3k_{B}T\Delta Q\left(pH\;\right)\Delta pH\;$$ (3)

As seen from Eq. 3, the complex stability change is directly proportional to the pH variation itself, ΔpH; it also depends on the pH of the environment *via* Δ*Q*(pH) = *Q*_*complex*_(pH) − *Q*_*unbound*_(pH). To utilize Eq. 3 for suggesting whether each calculated Δ*Q*(pH) may have biological consequences, we need three additional components: 1) the relevant pH of the environment; 2) ΔpH of biologically relevance; 3) a threshold of biological significance for the corresponding ΔΔG. Below is our reasoning for each of the three components.

1) One can easily argue that for chromatin components of eukaryotes, including the nucleosome and transcription factors, the most relevant pH is that inside the nucleus where the genetic material resides. Thus, unless otherwise stated, we assume pH = 7.4, which is likely close, to within a few decimal points, to mean intra-nuclear pH in higher organisms (Casey et al., 2009). Note that a more precise value of intra-nuclear pH is unimportant for us here because contribution to ΔΔG due to variation of Δ*Q*(pH) as a function of pH would appear only in the second order in ΔpH, and thus can be neglected in Eq. 3 for small enough ΔpH. We also assume that intra-nuclear pH ≈ intra-cellular pH, based on the common argument (Casey et al., 2009) that nuclear pores are large enough to allow virtually unimpeded passage of protons between the two compartments.

2) A more difficult issue is picking the specific ΔpH value most relevant to chromatin components, which should characterize typical intra-nuclear pH changes seen in living cells. We propose that the relevant ΔpH should be consistent with, and reflect the general magnitude of: natural cell-to-cell variations (Orij et al., 2012) of pH, its variation in response to external stimuli (Santos et al., 2016), shifts in intra-cellular pH seen in cancer cells (White et al., 2017) relative to normal ones, as well as temporal variations of intra-nuclear pH, e.g., related to the cell cycle progression (da Veiga Moreira et al., 2015). Based on the specific values of pH changes reported in these examples, we conservatively set the relevant ΔpH = 0.3, corresponding to a factor of 2 (*log*_10_2 ≈ 0.3) change in the proton activity of the solution. Note that we have deliberately excluded from the list the relatively large change in pH seen in apoptosis (Gottlieb et al., 1995), as we aim for the relevant ΔpH to be conservative and maximally inclusive. Once the relevant ΔpH is chosen, the corresponding complex stability change from Eq. 3 is ${\Delta \Delta G}_{pH}^{0.3}=2.3k_{B}T\Delta Q\left(pH\;\right)*0.3$ . Since our main interest is pH = 7.4, we will simplify the notation by dropping the lower index: ${\Delta \Delta G}^{0.3}\;={\Delta \Delta G}_{pH=7.4}^{0.3}$

3) Finally, we have to decide on the threshold of biological significance for ΔΔG^0.3^ in the context of chromatin function. One possible criterion (Fenley et al., 2018) is that, to be of potential biological significance, ΔΔG^0.3^ should be comparable with the free energy change characterizing some of the functionally relevant transitions in the given protein-nucleic acid complex. To proceed, we focus on chromatin associated protein-nucleic acid complexes. We need to decide on the smallest ΔΔG^0.3^ that can typically lead to meaningful biological consequences at the cellular level. One could make the usual physicist’s argument that ΔΔG^0.3^ values smaller than the thermal noise ∼ *k*_*B*_ *T* (∼ 0.6 kcal/mol) should be inconsequential. However appealing the “universal” 1*k*_*B*_ *T* threshold may appear, we argue that a more biologically motivated significance threshold for ΔΔG^0.3^ should be lower: $\frac{1}{2}k_{B}T$ , corresponding to $\sqrt{e}\approx 1.6$ fold change in the dissociation constant. The significance of the dissociation constant change of this magnitude can be argued for based on the fact that about 1.5 change in the transcription factor residence time can lead to a ∼3 -fold change in the transcription rate (mRNA molecules of the reporter gene) (Popp et al., 2021), which is significant. A similar argument is presented below in the context of the nucleosomal DNA accessibility. In what follows we assume the $\frac{1}{2}k_{B}T$ threshold of biological significance for predicted changes in the free energy of binding. To conclude this important part, we present a different argument for why the threshold for biological significance of ΔΔG^0.3^ may be fairly low. Let’s compare the proposed threshold to experimental binding affinities of chromatin-related complexes. A recent analysis of 83 protein-DNA complexes (Peng et al., 2021) revealed a broad distribution of experimentally measured complex binding affinities was observed, with the most likely value at $∼15k_{B}T$ ; the same study showed that the stability of *some* of them is less than 10 *k*_*B*_ *T*. Importantly, the experimental binding affinities referred to above are reported at the standard conditions, which implies 1M concentration. In reality, concentration of transcription factors *in vivo* can be (Milo and Phillips, 2015) as low as *nM* to *μ*M, which would reduce their relevant binding affinities by 12 to 8 *k*_*B*_ *T* from their standard state values, meaning that ΔΔG^0.3^ of even a fraction *k*_*B*_ *T* may affect the corresponding biological function of the protein-nucleic acid complex.

### 3.1 Twenty protein-nucleic acid complexes

**FIGURE 2:**
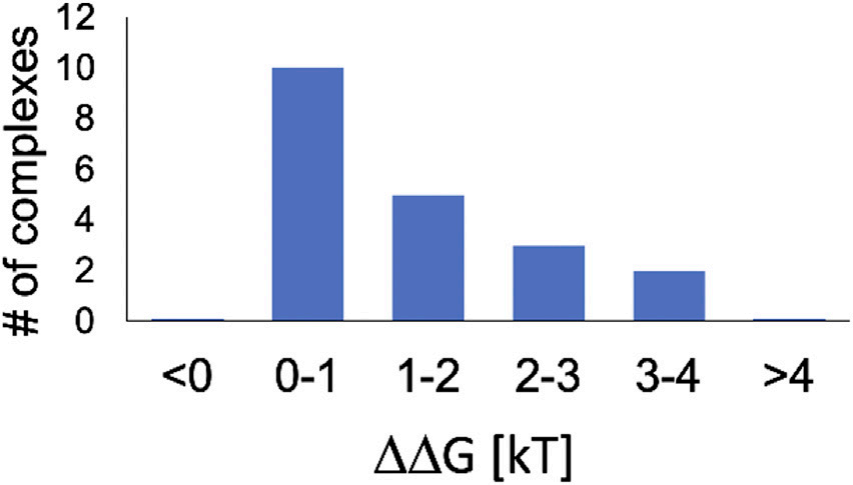
Most protein - nucleic acid complexes in the pH environment relevant to the nucleus are destabilized by increasing pH. Shown in the computed distribution of the change in complex stability, ΔΔG^0.3^, due to pH increase by 0.3 units. Within the [0, 1]*k*_*B*_ *T* interval, ΔΔG^0.3^is greater than the threshold of biological significance, $\frac{1}{2}k_{B}T$ , for 4 out of 10 complexes.

**FIGURE 3:**
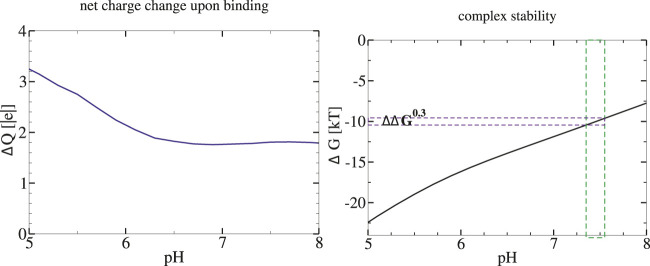
Stability of Methionine Repressor (MetJ) transcription factor complex with its DNA is predicted to decrease with pH of the environment. Left: pH dependence of the net charge change (proton uptake) upon complex formation. Right: Stability of the protein-DNA complex. The Δ*G*(pH) is estimated *via* Eq. 1 with Δ*G*(pH_*ref*_)=9.8*k*_*B*_ *T* (−5.8 kcal/mol (Hyre and Spicer, 1995)) at pH_*ref*_ =7.4. The vertical green bar around pH_*ref*_ indicates the proposed magnitude of biologically relevant variation of intra-nuclear pH; the corresponding ΔΔG^0.3^is also indicated.

Our main result for twenty, essentially random (see Methods for important details), protein-nucleic acid complexes is summarized in Figure 2. For biologically relevant variation of pH of 0.3 units, the corresponding change in the complex stability, ΔΔG^0.3^, varies widely, but for all of them ΔΔG^0.3^ ≥ 0. That is at pH = 7.4 relevant to typical intra-cellular conditions, all of the complexes are either unaffected or destabilized by increasing pH, meaning that the affinity between the protein and the nucleic acid decreases at higher pH; conversely, a drop in pH leads to stabilization of the complex. Moreover, we predict $\frac{\partial \Delta G\;}{\partial pH\;}\geq 0$ for all of the twenty complexes in the range from pH = 5 to 8, with $\frac{\partial \Delta G\;}{\partial pH\;}\approx 0$ for a minority of the complexes over only a portion of the range (results not shown). An illustrative example of this behavior for a transcription factor is discussed below, Figure 3.

Accepting the $\frac{1}{2}k_{B}T$ biological significance threshold suggested above, we find that 30% of the complexes fall below it. On the other hand, complex stability changes larger than $\frac{1}{2}k_{B}T$ occur for 70%, and changes larger than *k*_*B*_ *T* for 50% of the complexes; for 10% of them the effect is between 3 and 4 *k*_*B*_ *T*. We argue that some of these effects must have biological consequences.

As a specific example of our reasoning, consider the methionine repressor MetJ (PDB IDs 1MJM, 1MJK) from our set of twenty protein-nucleic acid complexes. This is a transcription factor, which represses the transcription of genes involved in methionine biosynthesis by binding to certain DNA sequences (Garvie and Phillips, 2000). In the cell, sensitivity of MetJ for the DNA is enhanced by binding of a specific co-repressor, resulting in a 10-fold increase in the binding constant of MetJ to the DNA (Saint-Girons et al., 1986). The computed proton uptake upon MetJ complex formation Δ*Q*(pH = 7.4) = 1.9, yielding ΔΔG^0.3^ = 1.3*k*_*B*_ *T* (0.77 kcal/mol). Therefore, a pH increase of 0.3, which reduces the intrinsic binding constant of MetJ to the DNA by a factor of 3.6, can lower the beneficial effect of the co-repressor binding significantly.

Relatively few experimental data points on pH dependence of protein-nucleic acid binding free energy are available, but every single one that we have identified so far corroborates our over-all conclusion that protein-nucleic acid complexes are destabilized with increasing pH. The experimental data points are briefly discussed below; direct comparison with the predicted ΔΔG^0.3^ is made where possible. In the list below, quantitative findings are discussed first, followed by more qualitative statements.

1. For the *λ*-repressor, the experimental ΔG(pH) curve (Senear and Ackers, 1990), shows progressive complex destabilization, $\frac{\partial \Delta G\;}{\partial pH\;}>0$ , over the entire “biologically relevant” range from pH = 5.0 to 8.0. Our prediction for the *λ*-repressor is the same, $\frac{\partial \Delta G\;}{\partial pH\;}>0$ over the same range of pH. Quantitatively, we predict ΔΔG^0.3^ = 1.1 *kT* for the *λ*-repressor, in reasonable agreement with the experimental (Senear and Ackers, 1990; Misra et al., 1998) ΔΔG^0.3^ = 0.77 ± 0.2*kT*, inferred from the experimental Δ*Q*(pH = 7.5) = 1 ± 0.2.
2. It was shown (Deegan et al., 2010) that the binding affinity of a specific transcription factor (estrogen nuclear receptor) decreases steadily with pH, the sharpest decrease occurring at around pH = 7.4; the over-all drop in the binding free energy is about 1.4*k*_*B*_ *T* over the range from pH = 7 to 8. From the reported experimental ΔG(pH) curve we estimate ΔΔG^0.3^ ≈ 0.5*k*_*B*_ *T*, which means that a biologically relevant variation in intra-cellular pH can change the receptor occupancy of the DNA binding site by a factor of ∼1.6 . By our own criterion, this effect is borderline significant for biological consequences; this complex was proposed to act as a pH sensor (Deegan et al., 2010). We did not perform p*K* and ΔΔG^0.3^ calculations for this complex because the available X-ray structure did not satisfy a single selection criterion used in this work, see “Methods.”

The five experimental data points below all confirm our general conclusion that protein-nucleic acid complex stability decreases with increasing pH.

• The binding activity of Sp1 protein (member of zinc finger family) to the CG box DNA linearly increased, 7-fold, as the pH was lowered from pH = 8.0 to 6.0. (Torigoe et al., 2003).
• A steady and significant decrease of the protein-DNA complex stability with increasing pH from 5 to 8 was observed experimentally for the complex of DNA with a GFP-bound protein (Moreau et al., 2012).
• Binding efficiency of vertebrate protein RDM1 for a 52-mer oligonucleotide gradually decreased as the pH was increased from 6.4, reaching near zero at pH = 7.0 (Hamimes et al., 2006).
• T-antigen (a specific DNA-binding protein from simian virus) binds with high affinity to unspecific DNA at pH = 6, with the affinity decreasing substantially at pH = 7.5 (Wessel et al., 1992).
• DNA-binding activity of polyomavirus large T-antigen decreases steadily from pH = 6.0 to 8.0 (Peng and Acheson, 1998).

From the linkage relationship, Eq. 3, the destabilization of protein-nucleic acid complexes with increasing pH occurs when the complex formation is followed by a net proton uptake, Δ*Q* = *Q*(*complex*) − *Q*(*unbound*) > 0. The physical origin of the net uptake is the attraction between the oppositely charged protons and the nucleic acid ligand, which favors protonated states of titratable groups of the bound protein at the given pH, or, equivalently, an increase in p*K* of these groups: p*K* (complex) > p*K* (unbound). Thus, for example, a negatively charged GLU or ASP is likely to pick up a proton from the solution and become neutral upon complex formation, while a LYS is more likely to be found positively charged in the complex then in the free protein. Essentially the same qualitative conclusion was made previously (Peng and Alexov, 2017), based on more than 300 protein-nucleic acid complexes. For the twenty complexes examined here, p*K* (complex) > p*K* (unbound) for the overwhelming majority, 95%, of the titratable groups, whether acidic or basic. However, in apparent contrast to Ref. (Peng and Alexov, 2017), we find a fairly substantial number of titratable groups from at least 2 to as many as 30, in each complex (an average of ∼ 12% of the total number of titratable groups per complex), for which the computed p*K* shifts are appreciable, p*K* (*complex*) − p*K* (*unbound*) > 1. We suggest^2^ that it is these larger p*K* shifts that explain the non-negligible Δ*Q*(*pH* = 7.4) > 0 seen for most of the complexes explored in this work, leading to potentially biologically relevant effect on the complex stability predicted for some of the complexes. We note several methodological differences with Ref. (Peng and Alexov, 2017), which concluded that Δ*Q*(*pH* = 7.4) ≈ 0 for many complexes. In particular, we used separately determined X-ray structures for the complex and the free (unligated) protein, where available. This computational procedure accounts for binding-induced structural re-arrangements (Ellis et al., 2016) in the protein, see “Methods” and Ref. (Aguilar et al., 2010), where it was demonstrated that substantial p*K* shifts were more pronounced when these structural re-arrangements were included in the computational protocol.

### 3.2 The nucleosome

Our first set of results in this section, Table 1, characterizes the predicted pH dependence of the nucleosome complex formation, that is the transition *DNA* + *histone octamer* → *nucleosome*.

**TABLE 1:** The effect of intra-nuclear pH variation of 0.3 units on the nucleosome stability can be substantial. Shown are the computed values of the reduction of the binding affinity between the DNA and the histone octamer due to pH increase by 0.3 units. The change is the binding affinity is expressed as ΔΔG^0.3^in units of *k*_*B*_ *T*. The predicted relatively strong effect is robust to the nucleosome structure (species) as well as the key parameter of the computation—the internal dielectric constant *ϵ*_*in*_. The *default* refers to *ϵ*_*in*_ =10 commonly used, while *ϵ*_*in*_ =4 is the lower bound on *ϵ*_*in*_ in proteins (Simonson, 2013).

	Organism		
Methodology	chimera	human	*xenopus*
default	10.2	9.2	8.6
*ϵ*_*in*_ = 4	16.4	13.0	11.1

Consistent with what we have seen for other protein-nucleic complexes, the nucleosome is destabilized by increasing pH. Conversely, a drop in intra-nuclear pH is predicted to have a stabilizing effect. Notably, the magnitude of the effect for ΔpH = 0.3, ΔΔG^0.3^ ∼ 10*k*_*B*_ *T* (6 kcal/mol), is considerably larger than that seen in all other protein-nucleic acid complexes examined so far, which probably reflects the key role of electrostatic interactions in the structure and function[13, 77 (Kunze and Netz, 2000; Beard and Schlick, 2001; Kunze and Netz, 2002; Schiessel, 2003),] of this complex, as well as the sheer length of its DNA—147 bp. The relatively large effect appears robust to the structure employed for the computation: the variation of the predicted ΔΔG^0.3^ between the different structures is within 2*k*_*B*_ *T*, or about 20%, for the default computational protocol, Table 1. Equally importantly, the estimate is robust to the key parameter[47, 48, 50] of the computational protocol—the internal dielectric constant. In fact, we can take the average difference between the ΔΔG^0.3^ in the 2nd and 3rd rows of Table 1 to arrive at a useful “error range” (Fenley et al., 2018) of ∼40 % on the computed ΔΔG^0.3^ for the nucleosome. In what follows we use ΔΔG^0.3^ ∼ 10*k*_*B*_ *T*, which corresponds to the bottom of that range.

Experimentally (Libertini and Small, 1984), the nucleosome core particle becomes looser as the pH of the environment is increased from 7 to 8, which can be seen as a qualitative confirmation of our result above. To the best of our knowledge, the free energy (stability) of the nucleosome complex as a function of pH has not been measured quantitatively, possibly because of the associated experimental difficulties (Thåström et al., 2004). We argue that the effect on the nucleosome stability of intra-cellular pH variations should be biologically relevant. First, the value of ΔΔG^0.3^ ∼ 10*k*_*B*_ *T* for the nucleosome is not negligible compared even to the high stability of the whole complex, which is about 64*k*_*B*_ *T* (38 kcal/mol) at *in-vivo* conditions (Fenley et al., 2010). An arguably more informative comparison of the ΔΔG^0.3^ is with changes of the binding affinity between the DNA and the histone octamer that occur due to various biologically relevant alterations of the nucleosome structure, which ultimately alter accessibility of its DNA. Note that a $\frac{1}{2}k_{B}T$ change in the protein-DNA binding affinity is enough to bring about a factor of 1.6 change in the probability of the DNA to be in the bound state. A change in the genomic DNA accessibility as small as a factor of 1.5, can have direct functional consequences such as transcription modulation, see, e.g*.*, a discussion in Ref. (Fenley et al., 2018). In particular, a number of charge-altering post-translational modifications (Tessarz and Kouzarides, 2014), or PTMs, directly modulate the strength of association between the histone octamer and nucleosomal DNA (Manohar et al., 2009; Materese et al., 2009; Andrews et al., 2010; Bowman and Poirier, 2015; Brehove et al., 2015). For example, acetylation of H3K56 in the globular core of the histone octamer destabilizes the nucleosome by ΔΔ*G* = 3.4*k*_*B*_ *T* (Andrews et al., 2010); this PTM, which increases DNA accessibility, was shown to increase transcription rates (Tessarz and Kouzarides, 2014). Thus, one can imagine a situation in which a drop in intra-nuclear pH of 0.3 units counterbalances the destabilizing effect of H3K56 thus reducing or completely reversing its effect on nucleosomal DNA accessibility and its functional consequences. In fact, most of lysine acetylations in the globular histone core, some of which are functionally relevant, are characterized (Fenley et al., 2018) by a decrease in DNA binding affinity of less than the nucleosomal ΔΔG^0.3^, which means that their effect on the genomic DNA accessibility can potentially be strongly modulated by biologically relevant intra-nuclear pH variations.

#### 3.2.1 Partial DNA unwrapping in the entry/exit region

Access to nucleosomal DNA can also be facilitated by spontaneous unwrapping of its end fragments (Tims et al., 2011), which can “peel off” transiently (Armeev et al., 2021). Here we have calculated ΔΔG^0.3^ that characterizes pH dependence of transient unwrapping 20 bp fragments at each end, see Fig. 1 (left) in “Methods.” The transient opening of this so-called “entry/exit” region (Fenley et al., 2018) can facilitate “invasion” of the nucleosome by key factors involved in initiating processes such as transcription (Tims et al., 2011). Our estimate is ΔΔG^0.3^ = 2.2*k*_*B*_ *T* for the entry/exit region sensitivity to pH. This magnitude of the ΔΔG^0.3^ is significantly higher than the proposed general threshold of biological significance, $\frac{1}{2}k_{B}T$ , corresponding to 1.6-fold change in the nucleosomal DNA accessibility (probability to be in the open state) (Fenley et al., 2018). Let’s explore more specific comparisons. The predicted ΔΔG^0.3^ for the entry/exit region corresponds to a 9-fold change in the nucleosomal DNA accessibility, which is much larger than the threshold of 1.5- fold change of biological significance proposed earlier (Fenley et al., 2018) for the entry/exit DNA accessibility change (Fenley et al., 2018). In particular, acetylation of H4K31, known to be associated with an order of magnitude increase in steady-state transcript levels (Zhu and Thiele, 1996), and promoter activity (Raveh-Sadka et al., 2012), leads to an estimated 1.5- fold change in the entry/exit DNA accessibility (Fenley et al., 2018). Based on the above, we make a specific prediction for most lysine acetylations that affect accessibility of the nucleosomal DNA in the entry/exit region: their effect can be significantly modulated by biologically relevant pH variations. These variations may negate, or significantly enhance, the effect of a specific acetylation on the DNA accessibility.

To conclude this subsection, let’s compare the free energy cost of unwrapping the entry/exit region to the corresponding ΔΔG^0.3^. The cost of unwrapping a ∼10 bp long DNA fragment at each end is $∼1.7k_{B}T$ (Koopmans et al., 2009; Wei et al., 2015), increasing roughly proportionally to the fragment length (Blossey and Schiessel, 2011; Culkin et al., 2017). Thus, the cost of unwrapping two 20 bp fragments is $∼6.8k_{B}T$ ; our predicted pH effect, ΔΔG^0.3^ = 2.2*k*_*B*_ *T*, is 1/3 of the total unwrapping cost, which is significant, further supporting the claim of the significance of the biologically relevant pH variations on the DNA accessibility in the entry/exit region of the nucleosome.

#### 3.2.2 Partially assembled states of the nucleosome

Yet another mechanism that facilitates access to the nucleosomal DNA is progressive disassembly of the histone octamer itself (Andrews and Luger, 2011; Luger et al., 2012), leading to the formation of partially assembled nucleosome structures, PANS (Rychkov et al., 2017). Among these, the tetrasome, (H3⋅H4)_2_ ⋅ DNA, Figure 1 (right), is believed to be highly relevant. Experimentally reported (Andrews et al., 2010) stability of the nucleosome with respect to this specific disassembly pathway, that is the free energy cost of removing H2A and H2B histones from the nucleosome to form the tetrasome, is $∼17k_{B}T$ . Assuming (Fenley et al., 2018) ∼300μM nucleosome concentration *in-vivo*, this value is reduced to about $∼9k_{B}T$ , see also the discussion above. Our estimate of the pH dependence of the (H3⋅H4)_2_ ⋅ DNA + (H2A⋅H2B)_2_ → *nucleosome* reaction, ΔΔG^0.3^ = 5.2*k*_*B*_ *T*, has comparable magnitude, implying that the nucleosome assembly/disassembly process can be modulated by biologically relevant small variations of intra-nuclear pH.

## 4 Conclusion

In this paper we have explored, computationally, the extent to which thermodynamic stability of protein-nucleic acid complexes may be affected by biologically relevant, small variations of ambient pH around its average value. Since our main interest is in protein-nucleic acid complexes relevant to chromatin function, the focus is on intra-nuclear/intra-cellular pH. We have argued, based on examples of pH variations reported for various biological processes, that a reasonable scale for the “biologically relevant” variation of intra-nuclear pH should be 0.3 units (around a mean value of pH ≈ 7.4). In some processes, e.g*., in apoptosis* (Gottlieb et al., 1995), experimentally reported relevant variation of pH were noticeably larger than 0.3, this is why we believe that our estimate of the significance threshold of 0.3 is conservative. The metric we have introduced to characterize the response of a protein-ligand complex to the change of ambient pH by 0.3 is the corresponding change, ΔΔG^0.3^, of the complex stability, or, equivalently, the change in the free energy of the protein-ligand binding, computed at pH = 7.4. In this work, estimation of ΔΔG^0.3^ for each complex utilized a set of standard computational tools and the fundamental thermodynamic linkage relationship that connects, exactly, the ΔΔG^0.3^ with the predicted net proton update (net charge change) upon complex formation.

We have computed ΔΔG^0.3^ for a set of twenty carefully selected protein-DNA and protein-RNA complexes that cover a range of biological functions. We have found that, in most cases, increasing pH leads to a drop in the binding affinity between the protein and nucleic acid, and *vice versa*. An intuitive, albeit very qualitative, explanation for this general trend is that the binding of a negatively charged nucleic acid can lead to some of the titratable groups of the receptor protein acquiring a proton due to its favorable charge-charge interaction with the oppositely charged ligand. The resulting net gain in the attractive free energy of the complex is higher when protons are more readily available in the ambient solution, that is at lower pH. Only for a small minority of the complexes examined here, it so happens that there is no net proton uptake upon nucleic acid binding around pH ∼ 7, and hence the complex stability does not depend on pH in this range. Further analysis has revealed a perhaps unexpected result: the effect of the relatively small pH variations can lead to non-negligible effect ΔΔG^0.3^ > 1/2*k*_*B*_ *T* (0.3 kcal/mol) on stability of about 70% of the diverse protein-nucleic acid complexes tested. And for at least 10% of the complexes the effect can be as large as 3 -4 *k*_*B*_ *T*. We argue for possible biological relevance of the variations of complex stability of 1/2*k*_*B*_ *T* or above, in the context relevant to main chromatin functions such as transcription and DNA replication. Note that a change in the protein-ligand binding affinity of just 1/2*k*_*B*_ *T* leads to a 1.6-fold change of the ligand occupancy, which is not insignificant. For a more quantitative argument, we have compared the ΔΔG^0.3^ with several known free energy scales characterizing some of the relevant transitions in the complexes of interest or the biological processes that depend on it. As an example, we have shown that binding of a specific transcription factor can be noticeably affected by a pH variation of 0.3 units. Comparison with available experimental data points, albeit not very many, corroborate our general conclusion regarding destabilization of protein-nucleic acid complexes with increasing pH.

We have applied the same computational approach and reasoning to the fundamental unit of DNA compaction in eukaryotes: the nucleosome. The biological function of the nucleosome is diverse, including packaging and protecting of the genomic DNA, as well as gene regulation (Kornberg and Lorch, 2020). Multiple structural transitions in the nucleosome system can change accessibility of its DNA to cellular machines, e.g., those involved in transcription or DNA replication. Increases in nucleosomal DNA accessibility as small as 1.5-fold can have significant biological consequences, e.g., up to 10-fold increase in transcription rate and promoter activity (Zhu and Thiele, 1996; Raveh-Sadka et al., 2012). These consequences of increased DNA accessibility are not sequence-specific, i.e., the effects are due to the increase of the DNA accessibility itself (Raveh-Sadka et al., 2012). Here we have explored several nucleosome core particle structures, considering the effect of pH on the stability of the entire complex between the histone octamer core and its DNA. We have also explored other possible structural transitions in the nucleosome that make its DNA more accessible to cellular machinery. In particular, we have considered the formation of a partially unwrapped state in which 20 base pairs “peel off” transiently at each end of the nucleosomal DNA, as well as a partially assembled state of the nucleosome (the tetrasome), where (H2A⋅H2B)_2_ dimer dissociates, exposing about 78 base pairs of the DNA.

We have compared the computed ΔΔG^0.3^ values with some of the biologically relevant energy scales in the above systems/transitions, and argued that ΔΔG^0.3^ is large enough to have biological significance. According to our predictions, the nucleosome complex is destabilized by about 10*k*_*B*_ *T* (6 kcal/mol) by a 0.3 unit increase in the ambient pH. The result is robust to a key parameter of the computational protocol and the specific atomistic structure employed for the computation. We have argued that, compared to relevant energy scales in the nucleosome, ΔΔG^0.3^ = 10*k*_*B*_ *T* is large enough to be biologically relevant. One such relevant energy scale is the change of the nucleosome stability due to charge-altering post-translation modifications (Fenley et al., 2018), many of which have been shown to lead to biologically relevant effects *in vivo*, e.g., increased transcription rates. Likewise, we predict that one of the key nucleosome disassembly pathways that exposes its DNA, *via* the tetrasome complex of four core histones with the DNA, is also sensitive to small pH variations, with ΔΔG^0.3^ = 5.2*k*_*B*_ *T* (3 kcal/mol). For spontaneous, transient unwrapping of 20 base pair long end fragments of the nucleosomal DNA, ΔΔG^0.3^ = 2.2*k*_*B*_ *T*. We have argued that this magnitude of sensitivity to ambient pH may also be relevant biologically.

The main limitation of the above quantitative analysis stems from the well known limitations of the underlying continuum solvent methodology we have employed for our ΔΔG^0.3^ estimates. Still, even a hypothetical factor of 2 downward error in our ΔΔG^0.3^ estimates should not invalidate our main conclusion that the pH effect on stability of the nucleosome, and on various other transitions that expose its DNA, is not insignificant compared to the relevant energy scales. Also note that our choice of biologically relevant ΔpH is quite conservative, as some biologically relevant processes are characterized by higher pH changes. For example, if we computed apoptosis-specific ΔΔG^0.3^ values, these would be about three times larger than those used to make the above conclusions, meaning that the predicted effect on the protein-nucleic acid stability would be about three times stronger. In particular, assuming ΔpH = 1.0 in Eq. 3, we obtain $∼33k_{B}T$ , $∼17k_{B}T$ , and $∼7k_{B}T$ for the corresponding stability change (increase), ΔΔG^0.3^, of the whole nucleosome, its dis-assmbly into the tetrasome, and unwrapping of the entry/exit region, respectively. These are large effects, diretly comparable, or even equal, to the corresponding free energies themselves, which means that the equilibrium is shifted strongly towards the intact, “frozen,” nucleosome away from its more “open” states. Accordingly, the drop in pH by 1 unit associated with apoptosis is predicted to lead to a very significant reduction of the nucleosomal DNA accessibility.

To use the above conclusions for making predictions *in-vivo* requires a leap of faith—faith both in simplicity and the power of reductionsim. Making the leap, we can predict that small, naturally occurring variations of intra-cellular pH can have detectable effect on processes that depend on the DNA accessibility in the nucleosome, e.g., transcription and replication. For example, we speculate that an increase in pH during the S-phase of the cell cycle (Putney and Barber, 2003) may upregulate the DNA replications rate; conversely, if this increase is somehow blocked, the cell cycle may elongate or halt altogether. Likewise, we speculate that both transcription and DNA replication should be upregulated in cancer cells because their intra-cellular pH is shifted upward relative to normal cells, meaning that the DNA association in the nucleosomes becomes relatively looser.^3^ Perhaps the strongest speculative prediction can be made for the effect of the significant pH drop seen in apoptosis. Assuming that the drop is an effector of the apoptosis program (Gottlieb et al., 1995), we predict significant downregulation of processess that depend on accessibility of the nucleosomal DNA, in particular DNA replication and transcription. The main limitation of this kind of reasoning is that the reality is likely more complex than the one assumed by our simplistic picture. For example, even considering only the results of this work, one can notice that, *e.g.*, transcription rate as a function of increased pH can be affected by two factors, working in the opposite directions: the more accessible DNA in the nucleosome is countered by the diminished effectiveness of transcription factor(s) due to their lower DNA binding affinity at a higher pH. A more complex, quantitative model may be required to make stronger connections between our ΔΔG^0.3^ predictions and what happens *in-vivo*. Going well beyond this work, it would be useful to establish whether the magnitude of biologically relevant intra-cellular pH variations are somehow “universal,” that is are not dissimilar between similar processes in higher eukaryotes. In our view, the following fact hints at this possibility: a large variety of different molecular mechanisms are involved in regulating intra-cellular pH, and these mechanisms appear highly redundant (Doyen et al., 2022). Tractable mathematical models (Doyen et al., 2022) can provide initial insights and guide future experiments. We suggest that the proposed “minimally relevant unit of pH variation” of 0.3, or equivalently, 2-fold change in the *H* ^+^ activity, may be adopted as a convenient threshold for classification of experimentally determined pH changes or differences as biologically relevant, at least in the context of chromatin function.

In summary, our predictions can be divided into two groups. First, we have made predictions that can be verified *in-vitro* by relatively standard biochemical methods such as isothermal titration calorimetry, which can revel the binding free energy. These measurements taken on a reasonably large sample of chromatin remodeling/transcription factors, at several points around pH = 7.4, can verify our main prediction that the pH effect on stability of these complexes is non-negligible, statistically speaking. In our opinion, pH dependence of the DNA accessibility in the nucleosome warrants a special investigation due to its potential importance. For example, pH dependence of spontaneous unwrapping of end fragments of nucleosomal DNA may potentially be tested within the same types of experiments (Gansen et al., 2009; Koopmans et al., 2009; Tims et al., 2011; Chen et al., 2014; Chen et al., 2017) that were used to investigate the original effect. Presumably, the same idea applies to experimental determination of pH dependence of the transitions involved in partial assembly/dis-assembly of the nucleosome (Andrews and Luger, 2011).

The second group of predictions includes the speculative connections to what may happen *in-vivo* as a result of small variations of intra-cellular pH. One may start to verify these connections by “minimal” assays, e.g., those that can test replication rates in nucleosomes arrays *in-vitro*. If these experiments do demonstrate meaningful pH dependence, then fully *in-vivo* experiment can begin to assess the most complex picture.

We conclude by proposing a curious, and potentially useful analogy between the nucleosome and the hydrogen atom. There are several layers to the analogy. First, this unique protein-DNA complex is the simplest fundamental unit of chromatin compaction in eukaryotes, its main building block. Second, by analogy with the electronic energy levels in the hydrogen atom, structural transitions in the nucleosome system are characterized by the appropriate energy scales, which determine which processes are allowed or forbidden. Further, the energy scales and the corresponding transitions can be affected by “external modulators”: fields, such as magnetic or electric field in the case of the real hydrogen atom or, as we have seen here, by pH in the case of the nucleosome. The list of the biologically relevant external modulators can be continued, potentially leading to further insights. For example, multiple species of mobile ions, including polyamines, are present in the nucleus; their relative abundances fluctuate, e.g., along the cell cycle. These ions can affect protein-nucleic acid binding *via* a variety of physical mechanisms, such as non-specific Debye screening of charge-charge interactions, and counterion-condensation effects. We suggest that the general approach advocated in this work should be applicable: the effect of each modulator can be quantified by the properly defined $\Delta \Delta G_{Y}^{X}$ , and compared with the biologically relevant variations of the modulator in question.

## Data Availability

Publicly available datasets were analyzed in this study. This data can be found here: https://www.rcsb.org/.

## Footnotes

1 Here we do not make a distinction between *pK*_*a*_ and p*K*_1/2_ (mid-point of titration curve), and denote both by p*K*, see, e.g., Ref (Onufriev et al., 2001). for an in-depth discussion of this issue.

2 For a single titratable site that experiences a small p*K* shift |ΔpK |≪ 1, the maximum proton uptake Δ*Q* ≈ 0.57ΔpK, at pH = p*K*. Therefore, if all of the p*K* shifts in the protein are small, no appreciable net proton uptake can be expected. Conversely, if at least some ΔpK >1 at pH ≈ p*K*, these sites will experience net proton uptake Δ*Q* ∼ 1, potentially leading to noticeable pH dependence of the complex stability.

3 In light of this, one may ask a hypothetical question what would be the effect of “alkaline diet” (sometimes advocated as an anti-cancer remedy), if the relevant pH could actually be altered by diet.
