# Supplementary material for: Biologically relevant small variations of intra-cellular pH can have significant effect on stability of protein–DNA complexes, including the nucleosome
Source: Front Mol Biosci. 2023 Apr 18;10:1067787. doi: 10.3389/fmolb.2023.1067787 (PMC10151541; doi:10.3389/fmolb.2023.1067787)
Supplement: Supplementary file 1 [file Table1.pdf]

Supplementary Material to:
Biologically relevant small variations of intra-cellular pH can have significant effect on stability of protein–DNA complexes, including the nucleosome.

1 THE TWENTY PROTEIN- NUCLEIC ACID COMPLEXES: ADDITIONAL INFORMATION.

Table S1. The data set of 20 protein-nucleic acid complexes. Functional annotation is inferred from the notes in the corresponding PDB file(s), or/and the primary reference(s) listed.

PDB ID of The complex	PDB ID of the Unligated protein	Ligand Type	Notes on Biological Function
3HDD	1ENH	DNA	TRANSCRIPTION REGULATION
1J59	1G6N	DNA	TRANSCRIPTION ACTIVATION
1M5O	1OIA	RNA	TRANSLATION
2E1C	2CYY	DNA	TRANSCRIPTIONAL REGULATOR
1MJM	1MJK	DNA	TRANSCRIPTION FACTOR, METHIONINE REPRESSOR
2AC0	2OCJ	DNA	P53 TUMOR SUPPRESSOR
1EMH	1AKZ	DNA	GLYCOSYLASE
1RCN	1C9X	DNA	RIBONUCLEASE
1RVA	1RVE	DNA	ENDONUCLEASE
2PVI	1K0Z	DNA	ENDONUCLEASE
1A74	1EVX	DNA	ENDONUCLEASE
1G9Z	2O7M	DNA	ENDONUCLEASE
1QUM	1QTW	DNA	ENDONUCLEASE
1G59	1GLN	RNA	GLUTAMYL-TRNA SYNTHETASE
2HW8	1AD2	RNA	RIBOSOMAL PROTEIN
1SDS	1RA4	RNA	MULTI-FUNCTIONAL, RIBOSOME ASSEMBLY
1M8W	1IB2	RNA	TRANSLATION REGULATION
1ZBL	1ZBF	RNA/DNA	RIBONUCLEASE H
2F8K	2D3D	RNA	POST-TRANSLATIONAL REGULATION
2A8V	1A62	RNA	TRANSCRIPTION TERMINATOR
